# Obstructive Salivary Gland Disorders - A Malaysian Patient Series

**DOI:** 10.1055/s-0044-1786833

**Published:** 2024-10-25

**Authors:** Sethu Thakachy Subha, Malina Osman, Prepageran Narayanan

**Affiliations:** 1 Department of Otorhinolaryngology, Faculty of Medicine and Health Sciences, Universiti Putra Malaysia (UPM), Serdang, Selangor, Malaysia; 2 Department of Microbiology, Faculty of Medicine and Health Sciences, Universiti Putra Malaysia (UPM), Serdang, Selangor, Malaysia; 3 Department of ENT, Faculty of Medicine, University Malaya, Kuala Lumpur, Malaysia

**Keywords:** risk factors, salivary gland disease, sialadenitis, sialolithiasis

## Abstract

**Introduction** Obstructive salivary gland disease is a frequently encountered clinical entity that can present to various health practitioners. Obstructive sialadenitis can lead to recurrent infections and debilitating quality-of-life issues.

**Objective** There is a paucity of published data regarding obstructive salivary disorders among the multiracial Asian population. The present study aimed to determine the demographic pattern and risk factors of obstructive salivary gland disorders with the goal of better management of this condition.

**Methods** A retrospective cross-sectional study was conducted at a tertiary institution over a period of 5 years.

**Results** A total of 256 (9.84 for every 1,000) patients were found to have salivary disorders, 10% of who were diagnosed to have obstructive disorder. Among the obstructive salivary disorders, 76% had sialolithiasis, 19% had recurrent parotitis, and 1 patient had chronic sialadenitis. We observed a small female preponderance for obstructive salivary disorders. This study revealed that smoking is a significant risk factor ( *p* = 0.041; prevalence ratio = 2.54, 95% confidence interval 1.12–5.78), and smokers were 2.5 times more likely to develop obstructive salivary disorders. There was no statistical correlation between the prevalence and other risk factors like infection, dehydration, intake of medications, history of diabetes mellitus, radiotherapy, and autoimmune disorders.

**Conclusion** Our study results demonstrated that the prevalence of obstructive salivary gland disorders was 0.1%. This study provided a better understanding of the prevalence and risk factors of obstructive salivary disorders, which facilitate timely management and improves quality of life.

## Introduction

Obstructive salivary gland disease represents the commonest inflammatory disorder of major salivary glands. The wide range of clinical presentations and the limitations in conventional imaging modalities involved in obstructive salivary disorders pose challenges in diagnosis and management. Among the salivary gland diseases, the most frequent non-neoplastic salivary disorder is obstructive sialadenitis, which may be due to calculi, mucous plugs, duct stenosis, kinks, stricture foreign bodies, anatomic variations, or malformations of the salivary duct system. 1 2

Sialolithiasis is the commonest cause of obstructive sialadenitis, accounting for ∼ 50% of major salivary gland diseases. 3 4 Obstructive salivary gland disorders can progress to acute sialadenitis with high fever, severe pain, and increased swelling as well as recurrent or chronic sialadenitis. 1 5 6 When left untreated, obstructive salivary disorder can cause debilitating quality-of-life issues. As such, in Malaysia and other Southeast Asian countries, there are hardly any published data regarding obstructive salivary disorders. Hence, it is important to study the demographic distributions of the patients and to determine risk factors and types of management of patients with obstructive salivary disorders. The association between demographics and the risk factors among patients with obstructive salivary disorders ensures better management can be provided once the factors are discerned.

## Methods

This is a retrospective cross-sectional study conducted at the otorhinolaryngology department of the selected tertiary institution in Malaysia. The following details of the patients were retrieved from the hospital registry: age, gender, general medical history, family history, habits, clinical symptoms, investigations, diagnosis, and management. All local citizens with a confirmed diagnosis of obstructive salivary gland disorders, presented at the otorhinolaryngology department from 1st January 2015 to 31st December 2019, were included in this study. Ethical approval was obtained from the Medical Research Ethics Committee of the tertiary center (MREC ID NO 2109611–7508).

Statistical analysis was performed using the IBM SPSS Version 25.0 software (IBM Corp., Armonk, NY, USA). A *p* -value less than 0.05 was considered significant. Descriptive continuous variables were presented according to findings in the normality test; further various analysis tests were used to determine the association between factors and the presence of salivary disorders.

## Results

A total of 256 patients (9.84 for every 1,000 patients) during the 5 years were found to have salivary disorders. Out of these, 26 (10.2%) were diagnosed to have obstructive salivary disorders. The overall prevalences of salivary disorders and obstructive salivary disorders for this study were 10% and 0.1%, respectively. The frequency distribution of non-obstructive salivary disorders included parotitis 115 (45%), sialadenitis 45 (18%), salivary abscess 41 (16%), non-specific salivary swellings 17 (13%), benign tumors 8 (3%), and malignant tumors 4 (1%).

Our demographic analysis demonstrated that the age range of the affected patients varies from 1 to 97 years with a median (interquartile range - IQR) of 38.0 (39.5). There was a higher proportion of obstructive salivary disorders among those aged 60 and above. However, the study did not reveal any significant association between age group and obstructive salivary gland diseases ( *p* = 0.53).

Among our study group, the descriptive analysis showed more females are affected by obstructive salivary disorders when compared with males. However, there is no statistically significant association between the prevalence and gender, as shown in Table 1 .

**Table 1:** Distribution of subjects according to demographic data and identified risk factors related to salivary disorder

Risk factors	Obstructive salivary gland disorder n (%)	Non-obstructive salivary gland disorder n (%)	Total n	ᵡ ^2^	*p*	Prevalence ratio (95% CI)
**Gender**				0.492	0.483	
Male	11 (8.8)	114 (91.2)	125			
Female	15 (11.5)	116 (88.5)	131			
**Race**				2.319	0.314	
Malay	14 (9.3)	137 (90.7)	151			
Chinese	5 (8.2)	56 (91.8)	61			
Indian	7 (16.7)	35 (83.3)	42			
Infection				1.99	0.274	−
Yes	16 (12.2)	115 (87.8)	131			
No	10 (8.1)	114 (91.1)	124			
Dehydration				0.981	0.322	−
Yes	6 (7.4)	75 (92.6)	81			
No	20 (11.4)	155 (88.6)	175			
Diabetes mellitus				0.973	0.324	−
Yes	8 (13.6)	51 (86.4)	59			
No	18 (19.1)	179 (90.9)	197			
Hypothyroidism				0.461	0.497	−
Yes	0 (0)	4 (100)	4			
No	26 (10.4)	225 (89.6)	251			
GERD				1.29	0.254	−
Yes	0 (0)	11 (100)	11			
No	26 (10.6)	219 (89.4)	245			
Smoking					0.041*	2.54 (1.12–5.78)
Yes	6 (22.2)	21 (77.8)	27			
No	20 (8.7)	209 (91.3)	229			
Smoking **					0.029*	2.62 (1.11–6.17)
Yes	6 (22.2)	21 (77.8)	27			
No	15(8.5)	162 (91.5)	177			
Alcohol					1.00	
Yes	0 (0)	8 (100)	8			
No	26 (10.5)	222 (89.5)	248			
Radiotherapy					0.276	
Yes	1 (33.3)	2 (66.7)	3			
No	25 (9.9)	228 (90.1)	253			
Medication					0.703	
Yes	1 (5.3)	18 (94.7)	19			
No	25 (10.5)	212 (89.5)	237			
Previous surgery			256			
Yes	0 (0)	0 (0)				
No	26 (10.2)	230 (89.8)				
Autoimmune					0.349	
Yes	2 (16.7)	10 (83.3)	12			
No	24 (9.8)	220 (90.2)	244			

** adult population (18 years and above).

Regarding ethnicity, most of the patients (53.8% [14]) were Malays, followed by Chinese (19.2% [5]), and Indians (27% [7]), which represents the local demographic data. Our statistical analysis did not show any significant association between ethnicity and prevalence.

Of the obstructive group patients, 22.2% (6/26) were documented to be smokers. Our study demonstrated that those who smoked were 2.54 times more likely to develop obstructive salivary disorder, and smoking was found to be significantly correlated to the prevalence ( *p* = 0.041, prevalence ratio = 2.54, 95% confidence interval [CI] = 1.12–5.78). The impact of smoking on the prevalence of obstructive salivary disorders among the adult population (18 years and above) has shown a significant correlation ( *p* = 0.029, pre prevalence ratio = 2.62, 95% CI = 1.11–6.17).

The correlation between other potential risk factors in the literature and prevalence was analyzed and found to be insignificant. Although the infection rate was 12% (16/26), there was no statistical correlation between the infection and the prevalence. Concerning other risk factors, such as dehydration (7%), intake of medications (5%), history of diabetes mellitus (13%), radiotherapy (33%), and autoimmune disorders (16%), there was also no significant correlation between these and the prevalence rate.

The analysis revealed that none of our sample group of patients had other risk factors like a history of previous surgery, hypothyroidism, and gastroesophageal reflux disease.

The common etiology of our obstructive salivary disorders was sialolithiasis (76% [20/26]) followed by recurrent parotitis with abscess (19% [5/26]), and chronic sialadenitis (4% [1/26]).

Regarding the overall patients with salivary disorders, 76% of our patients underwent conservative management. Out of 26 patients with obstructive salivary disorders, 15 (38%) underwent calculi removal by intraoral approach, and none of our patients had a sialendoscopy procedure.

Three patients (11%) had gland removal and seven were treated conservatively only with antibiotics. Eight patients received combined treatment.

## Discussion

Our retrospective study was conducted at a selected tertiary hospital among the population of ear, nose, and throat clinic patients over a period of 5 years. The present study demonstrated the prevalence of obstructive salivary disorders is around 0.1%, and the common etiology was sialolithiasis (76.9%). This is similar to the findings of studies by Holden et al. and Escudier et al., with sialolithiasis accounting for 66% of cases with a symptomatic incidence of 27 to 59 cases per million per annum. 6 7 Escudier et al. and McGurk et al. reported that the clinical prevalence of symptomatic salivary calculi was 0.45%, 7 8 whereas Raunch et al., in their postmortem studies, have shown a 1.2% prevalence of salivary calculi in the general population. 9

Among our study patients, we found a small female preponderance and a higher proportion above 60 years of age for obstructive salivary disorders. This is in accordance with the meta-analysis by Jadu et al., which reported the peak incidence of sialolithiasis in the fourth to sixth decades and more common in females. 10 This was in contrast to other studies which reported that sialolithiasis is comparatively more common in male patients with a mean age of 45 years. 11 12 A few other studies have shown that salivary stones can occur in an almost equal distribution between males and females. 13

In general, the etiology of sialadenitis can be either infectious or non-infectious. 14 The infective factors of acute sialadenitis can be bacterial or viral. The proposed mechanisms of inflammation are retrograde infection secondary to reduced salivary flow or obstruction in the duct. 14 Sialolithiasis and ductal strictures are the commonest causative factors that can diminish the salivary flow and lead to acute, chronic, or recurrent infections. 15 16 17 18 This is in agreement with the result of our study, in which 76.9% of obstructive salivary disorder patients had sialolithiasis. The literature has shown that around 80 to 90% of sialolithiasis occurs in the submandibular salivary gland followed by the parotid and sublingual salivary gland. The exact mechanism of sialolithiasis is not known. The decreased salivary flow or formation, inflammation and retrograde infection have been proposed as the associated factors to the development of sialolithiasis. 15 18 19 Numerous studies indicated that some of the risk factors for decreased salivary flow can be dehydration, tobacco smoking, certain medications with anticholinergic properties, debilitating conditions like diabetes mellitus, hypothyroidism and renal failure, Sjögren syndrome, and postradioiodine therapy. 15 16

Our study revealed a significant statistical correlation between the prevalence of obstructive salivary disorders and smoking. A clinical study by Rad et al. showed that the long-term effects of tobacco use and persistent smoking lead to a reduced salivary flow rate and may contribute to sialolithiasis. 20 Tobacco smoking has also been linked to diminished salivary antimicrobial activity, which makes the salivary ducts and glands susceptible to infection and inflammation. 13 20 Huoh et al. reported a higher rate of smoking in patients with salivary stones than in the general population, which was not statistically significant. 16 Kraaij et al., in their study, also observed smoking increases the risk of developing a salivary stone but without a statistically significant correlation between salivary stone formation, smoking, and alcohol consumption. 13 Klein et al., in their retrospective study, found that there was no correlation between alcohol consumption and obstructive salivary gland pathologies. 21

Various studies revealed an increased risk of developing sialadenitis in patients with dehydration, malnutrition, poorly controlled diabetes, kidney diseases, and those who had received radiotherapy. 17 22 A retrospective case-control study by Kraaij et al. found that systemic diseases, medication, smoking, and alcohol consumption play a very limited role in the onset of salivary stones. 13 Similar to that study, statistical analysis of the risk factors in our cohort did not reveal any significant correlation between these and the prevalence rate of obstructive salivary disorders. Reports have shown that sialadenitis can be predisposed by medications like anticholinergics, diuretics, and β-blockers. 23 24

Ultrasound, standard and cone-beam computed tomography and magnetic resonance sialography are the imaging tools for the diagnosis of obstructive salivary disorders. 1 25 Currently the imaging has shifted away from conventional sialography to ultrasound, computed tomography (CT), magnetic resonance imaging (MRI), and MRI-sialography. Ultrasound is considered the first-line imaging modality in the detection of sialolithiasis and inflammatory salivary disorders. 26 27 Magnetic resonance imaging -sialography is an excellent non-invasive imaging to characterize the ductal structure of the parotid and submandibular glands. 28

The current management involves treating the acute infection, followed by definitive treatment of the causative factors. 18 This includes administration of antibiotics, sialagogues, hydration, and oral hygiene. Surgical drainage is indicated when acute sialadenitis is complicated with abscess formation. If the obstruction is due to calculi, these must be surgically removed once the infection is resolved.

The introduction of sialendoscopy in obstructive sialadenitis has created a paradigm shift in its management. 26 This minimally invasive approach can be utilized as both a diagnostic and therapeutic tool. Sialendoscopy can be considered as the first-line therapy for most ductal obstructive pathologies like sialolithiasis, ductal stenoses, etc. 27 Combined approaches involving sialendoscopy with the transoral or transcutaneous routes can be implemented in the management of obstructive salivary disorders. 1 21 24 Extra-corporeal shock-wave lithotripsy (ESWL) is a non-invasive method of fragmenting salivary stones into smaller portions to favor their possible flushing out from the salivary duct system. 8 30

## Conclusion

Obstructive salivary disorder is a relatively common problem with diverse clinical presentations. This study provides a better understanding of the prevalence and risk factors of obstructive salivary disorders. This will enable the timely detection and appropriate management of patients with this condition and improve their quality of life. Further prospective studies need to be done to evaluate the various types of management in non-neoplastic salivary disorders.

## Limitations

The data for this study is collected from only one hospital, and, thus, the data collected might not represent the general population of patients with obstructive salivary gland disorders. Therefore, the results of the study are likely to represent an underestimate of the prevalence of obstructive salivary gland disorders. Furthermore, this research is a retrospective cohort study using the existing records for data collection. Hence, there might be some absent details in the existing records, lowering the quality of the present data.
